# Alveolar Macrophage Chromatin Is Modified to Orchestrate Host Response to *Mycobacterium bovis* Infection

**DOI:** 10.3389/fgene.2019.01386

**Published:** 2020-02-07

**Authors:** Thomas J. Hall, Douglas Vernimmen, John A. Browne, Michael P. Mullen, Stephen V. Gordon, David E. MacHugh, Alan M. O’Doherty

**Affiliations:** ^1^Animal Genomics Laboratory, UCD School of Agriculture and Food Science, College Dublin, Dublin, Ireland; ^2^The Roslin Institute and Royal (Dick) School of Veterinary Studies, University of Edinburgh, Easter Bush, Midlothian, United Kingdom; ^3^Bioscience Research Institute, Athlone Institute of Technology, Athlone, Ireland; ^4^UCD School of Veterinary Medicine, University College Dublin, Dublin, Ireland; ^5^UCD Conway Institute of Biomolecular and Biomedical Research, University College Dublin, Dublin, Ireland

**Keywords:** ChIP-seq, chromatin, integrative genomics, macrophage, microRNA-seq, *Mycobacterium bovis*, RNA-seq, tuberculosis

## Abstract

Bovine tuberculosis is caused by infection with *Mycobacterium bovis*, which can also cause disease in a range of other mammals, including humans. Alveolar macrophages are the key immune effector cells that first encounter *M. bovis* and how the macrophage epigenome responds to mycobacterial pathogens is currently not well understood. Here, we have used chromatin immunoprecipitation sequencing (ChIP-seq), RNA-seq and miRNA-seq to examine the effect of *M. bovis* infection on the bovine alveolar macrophage (bAM) epigenome. We show that H3K4me3 is more prevalent, at a genome-wide level, in chromatin from *M. bovis*-infected bAM compared to control non-infected bAM; this was particularly evident at the transcriptional start sites of genes that determine programmed macrophage responses to mycobacterial infection (e.g. M1/M2 macrophage polarisation). This pattern was also supported by the distribution of RNA Polymerase II (Pol II) ChIP-seq results, which highlighted significantly increased transcriptional activity at genes demarcated by permissive chromatin. Identification of these genes enabled integration of high-density genome-wide association study (GWAS) data, which revealed genomic regions associated with resilience to infection with *M. bovis* in cattle. Through integration of these data, we show that bAM transcriptional reprogramming occurs through differential distribution of H3K4me3 and Pol II at key immune genes. Furthermore, this subset of genes can be used to prioritise genomic variants from a relevant GWAS data set.

## Introduction

Bovine tuberculosis (bTB) is a chronic infectious disease of livestock, particularly domestic cattle (*Bos taurus*, *Bos indicus* and *Bos taurus*/*indicus* hybrids), which causes more than $3 billion in losses to global agriculture annually (Steele, 1995; Waters et al., 2012). The aetiological agent of bTB is *Mycobacterium bovis*, a pathogen with a genome sequence that is 99.95% identical to *M. tuberculosis*, the primary cause of human tuberculosis (TB) (Garnier et al., 2003). In certain agroecological milieus *M. bovis* can also cause zoonotic TB with serious implications for human health (Thoen et al., 2016; Olea-Popelka et al., 2017; Vayr et al., 2018).

Previous studies have shown that the pathogenesis of bTB disease in animals is similar to TB disease in humans and many of the features of *M. tuberculosis* infection are also characteristic of *M. bovis* infection in cattle (Waters et al., 2014; Buddle et al., 2016; Williams and Orme, 2016). Transmission is *via* inhalation of contaminated aerosol droplets and the primary site of infection is the lungs where the bacilli are phagocytosed by alveolar macrophages, which normally can contain or destroy intracellular bacilli (Weiss and Schaible, 2015; Kaufmann and Dorhoi, 2016). Disease-causing mycobacteria, however, can persist and replicate within alveolar macrophages *via* a bewildering range of evolved mechanisms that subvert and interfere with host immune responses (de Chastellier, 2009; Cambier et al., 2014; Schorey and Schlesinger, 2016; Awuh and Flo, 2017). These mechanisms include recruitment of cell surface receptors on the host macrophage; blocking of macrophage phagosome–lysosome fusion; detoxification of reactive oxygen and nitrogen intermediates (ROI and RNI); harnessing of intracellular nutrient supply and metabolism; inhibition of apoptosis and autophagy; suppression of antigen presentation; modulation of macrophage signalling pathways; cytosolic escape from the phagosome; and induction of necrosis, which leads to immunopathology and shedding of the pathogen from the host (Ehrt and Schnappinger, 2009; Hussain Bhat and Mukhopadhyay, 2015; Queval et al., 2017; BoseDasgupta and Pieters, 2018; Chaurasiya, 2018; Stutz et al., 2018).

Considering the dramatic perturbation of the macrophage by intracellular mycobacteria, we and others have demonstrated that bovine and human alveolar macrophage transcriptomes are extensively reprogrammed in response to infection with *M. bovis* and *M. tuberculosis* (Nalpas et al., 2015; Vegh et al., 2015; Lavalett et al., 2017; Jensen et al., 2018; Malone et al., 2018; Papp et al., 2018). These studies have also revealed that differentially expressed gene sets and dysregulated cellular networks and pathways are functionally associated with many of the macrophage processes described above that can control or eliminate intracellular microbes.

For many intracellular pathogens, it is now also evident that the infection process involves alteration of epigenetic marks and chromatin remodelling that may profoundly alter host cell gene expression (Hamon and Cossart, 2008; Bierne et al., 2012; Rolando et al., 2015; Niller and Minarovits, 2016). For example, distinct DNA methylation changes are detectable in macrophages infected with the intracellular protozoan *Leishmania donovani*, which causes visceral leishmaniasis (Marr et al., 2014). Recent studies using cells with a macrophage phenotype generated from the THP-1 human monocyte cell line have provided evidence that infection with *M. tuberculosis* induces alterations to DNA methylation patterns at specific inflammatory genes (Zheng et al., 2016) and across the genome in a non-canonical fashion (Sharma et al., 2016).

With regards to host cell histones and in the context of mycobacterial infections, Yaseen et al. (2015) have shown that the Rv1988 protein, secreted by virulent mycobacteria, localises to the chromatin upon infection and mediates repression of host cell genes through methylation of histone H3 at a non-canonical arginine residue. In addition, chromatin immunoprecipitation sequencing (ChIP-seq) analysis of H3K4 monomethylation (a marker of poised or active enhancers) showed that regulatory sequence motifs embedded in subtypes of Alu SINE transposable elements are key components of the epigenetic machinery modulating human macrophage gene expression during *M. tuberculosis* infection (Bouttier et al., 2016).

In light of the profound macrophage reprogramming induced by mycobacterial infection, and previous work demonstrating a role for host cell chromatin modifications, we have used ChIP-seq and RNA sequencing (RNA-seq) to examine gene expression changes that reflect host–pathogen interaction in bovine alveolar macrophages (bAM) infected with *M. bovis*. The results obtained support an important role for dynamic chromatin remodelling in the macrophage response to mycobacterial infection, particularly with respect to M1/M2 polarisation. Genes identified from ChIP-seq and RNA-seq results were also integrated with genome-wide association study (GWAS) data to prioritise genomic regions and single-nucleotide polymorphisms (SNPs) associated with bTB resilience. Finally, the suitability of bAM for ChIP-seq assays and the results obtained demonstrate that these cells represent an excellent model system for unravelling the epigenetic and transcriptional circuitry perturbed during mycobacterial infection of vertebrate macrophages.

## Materials and Methods

### Preparation and Infection of bAM

bAM and *M. bovis* 2122 were prepared as described previously (Magee et al., 2014) with minor adjustments. Macrophages (2 × 10^6^) were seeded in 60 mm tissue culture plates and challenged with *M. bovis* at a multiplicity of infection (MOI) of 10:1 (2 × 10^7^ bacteria per plate) for 24 h; parallel non-infected controls were prepared simultaneously.

### Preparation of Nucleic Acids for Sequencing

Sheared fixed chromatin was prepared exactly as described in the truChIP™ Chromatin Shearing Kit (Covaris) using 2 × 10^6^ macrophage cells per AFA tube. Briefly, cells were washed in cold PBS and 2.0 ml of Fixing Buffer A was added to each plate, to which 200 µl of freshly prepared 11.1% formaldehyde solution was added. After 10 min on a gentle rocker the crosslinking was halted by the addition of 120 µl of Quenching Solution E; cells were washed with cold PBS, released from the plate using a cell scraper and resuspended in 300 µl Lysis Buffer B for 10 min with gentle agitation at 4°C to release the nuclei. The nuclei were pelleted and washed once in Wash Buffer C and three times in Shearing Buffer D3 prior to being resuspended in a final volume of 130 µl of Shearing Buffer D3. The nuclei were transferred to a micro AFA tube and sonicated for 8 min each using the Covaris E220e as per the manufacturer’s instructions. Chromatin immunoprecipitation of sonicated DNA samples was carried out using the Chromatin Immunoprecipitation (ChIP) Assay Kit (Merck KGaA) and anti-H3K4me3 (05-745R) (Merck KGaA), Pol II (H-224) (Santa Cruz Biotechnology, Inc.) or anti-H3K27me3 (07-449) (Merck KGaA) as previously described (Vernimmen et al., 2011). RNA was extracted from infected (*n* = 4) and control (*n* = 4) bAM samples using the RNeasy Plus Mini Kit (Qiagen) as previously described (O'Doherty et al., 2012). All eight samples exhibited excellent RNA quality metrics (RIN >9).

### Sequencing

Illumina TruSeq Stranded mRNA and TruSeq Small RNA kits were used for mRNA-seq and small RNA-seq library preparations and the NEB Next Ultra ChIPseq Library Prep kit (New England Biolabs) was used for ChIP-seq library preparations. Pooled libraries were sequenced by Edinburgh Genomics (http://genomics.ed.ac.uk) as follows: paired-end reads (2 × 75 bp) were obtained for mRNA and ChIP DNA libraries using the HiSeq 4000 sequencing platform and single-end read (50 bp) were obtained for small RNA libraries using the HiSeq 2500 high output version 4 platform.

### ChIP-seq Bioinformatics Analysis

Computational analyses for all bioinformatic processes were performed on a 72-CPU computer server with Linux Ubuntu (version 16.04.4 LTS). An average of 54 M paired end 75 bp reads were obtained for each histone mark. At each step of data processing, read quality was assessed *via* FastQC (version 0.11.5) (Andrews, 2016). Any samples that indicated adapter contamination were trimmed *via* Cutadapt (version 1.15) (Martin, 2011). Correlation plots generated with EaSeq (version 1.05) (Lerdrup et al., 2016) of genome-wide H3K4me3, H3K27me3 and Pol II sequencing reads from infected and non-infected bAM showed high correlation between samples (Pearson’s correlation coefficient: 0.93–0.97) for all three ChIP-seq targets (**Supplementary Figure 1**). After data quality control and filtering, ~760 million paired end reads were aligned to the UMD 3.1 bovine genome assembly using Bowtie2 (version 2.3.0) (Langmead and Salzberg, 2012). The mean alignment rate for the histone marks was 96.23%. The resulting SAM files were converted and indexed into BAM files *via* Samtools (version 1.3.1) (Li et al., 2009). After alignment, samples were combined and sorted into 14 files, based on the animal (A1 or A2), the histone mark (K4/K27/Pol II) and treatment (control or infected), i.e. A1-CTRL-K4. Peaks were called by using alignment files to determine where the reads have aligned to specific regions of the genome, and then comparing that alignment to the input samples as a normalisation step.

The peak calling was carried out *via* MACS2 (version 2.1.1.20160309) (Feng et al., 2011). The H3K4me3 mark was called in sharp peak mode and H3K27me3 and Pol II were called in broad peak mode, as per the user guide. Peak tracks were generated in MACS2 and visualised with the Integrative Genome Viewer (version 2.3) (Thorvaldsdottir et al., 2013). Union peaks were generated by combining and merging overlapping peaks in all samples for each histone mark. Differential peak calling was called *via* MACS2 using the bdgdiff function. Peak images were generated by visually assessing all three marks in tandem across the entire bovine genome with the Integrative Genomics Viewer (IGV). The significance of peaks was determined by sorting peaks for each mark in each treatment by *P* value and then fold enrichment with a cut-off of 2.0 and a *P* value threshold of 0.05 (Wilbanks and Facciotti, 2010). Peaks from each animal in each condition for each mark were cross-referenced with the IGV images and differential peak caller to determine a difference in fold enrichment for each observed peak difference between conditions. This required comparing peak start and end sites, chromosomes, *P* and *q* values for each summit, summit locations and normalised fold enrichment of a peak against the input sample (see **Supplementary Information File 1** for peak sets). Any peaks that exhibited a difference of 4 or greater fold enrichment, a *P* value of less than 0.05, a false discovery rate (FDR) less than 0.05 and that were also identified by the differential peak caller were selected for further analysis [see **Supplementary Information File 1** for peaks at transcription start sites (TSSs) that met some but not all of the above criteria]. Peaks that were then classified to be different between conditions in all three data sets were examined to determine their proximity to TSS. Differential peaks were also called using the R package DiffBind (version 2.80) (Stark and Brown, 2011). DiffBind includes functions to support the processing of peak sets, including overlapping and merging peak sets, counting sequencing reads overlapping intervals in peak sets and identifying statistically significantly differentially bound sites based on evidence of binding affinity (measured by differences in read densities; see **Supplementary Information File 1**). For H3K27me3 DiffBind differential peak calling, the initial MACS2 peak list, consisting of 64,264 total peaks (see **Supplementary Information File 1**), was merged and reduced to a smaller group of larger, broader peaks to reduce noise and false positive discovery (**Figure 2B**).

### RNA-Seq Bioinformatics Analysis

An average of 44 M paired end 75 bp reads were obtained for each of the eight samples (four control, four infected). Adapter sequence contamination and paired-end reads of poor quality were removed from the raw data. At each step, read quality was assessed with FastQC (version 0.11.5). Any samples that indicated adapter contamination were trimmed *via* Cutadapt (version 1.15). After quality control and filtering, ~250 million reads were mapped to the bovine genome, with 72% total read mapping, overall. The raw reads were aligned to the UMD 3.1.1 bovine transcriptome using Salmon (version 0.8.1) (Patro et al., 2017). Aligned reads were also counted in Salmon and the resulting quantification files were annotated at gene level *via* tximport (version 3.7) (Soneson et al., 2015). The annotated gene counts were then normalised and differential expression analysis performed with DESeq2 (version 1.20.0) (Love et al., 2014), correcting for multiple testing using the Benjamini–Hochberg method (Benjamini and Hochberg, 1995). Genes identified from ChIP-seq as exhibiting differential histone modifications were cross-referenced with the RNA-seq data set to determine significant log_2_FC between *M. bovis*-infected and control non-infected. Additionally, this RNA-seq data was cross-referenced with RNA-seq data from a previous study that investigated bAM infected with *M. bovis* (Nalpas et al., 2015).

### MicroRNA-Seq Bioinformatics Analysis

A mean of 26 M paired-end 50 bp reads were obtained for each of the eight samples (four control, four infected). At each step of data processing, read quality was assessed *via* FastQC (version 0.11.5). Any samples that exhibited adapter contamination were trimmed *via* Cutadapt (version 1.15) and all reads smaller than 17 bp were removed from the analysis. After quality control and filtering, ~100 million reads were mapped to the bovine genome, with 79% total reads mapping, overall. Raw reads were mapped to UMD3.1 using Bowtie (version 1.2.2). miRNA detection, identification and quantification were carried out with mirdeep2 (version 0.0.91). Isoform analysis was also performed using mirdeep2. Differential expression analysis was performed using DESeq2, correcting for multiple testing with the Benjamini–Hochberg method. Any miRNAs that were significantly differentially expressed (FDR < 0.10) were selected for further analysis. To determine if significantly differentially expressed miRNAs target genes selected in the ChIP-seq analysis, miRmap (Vejnar and Zdobnov, 2012) was used to predict the likelihood that a specific miRNA targets one or more of the genes based on three criteria: delta G binding, probability exact and phylogenetic conservation of seed site, which is then combined into a single scoring metric (miRmap score). Any predicted gene targets with miRmap score ≥0.70 were included in the analysis (see **Supplementary Information File 3**).

### Pathway Analysis

Pathway analysis was carried out on any gene that had a differential peak between control and infected samples. Pathway analysis and gene ontology (GO) summarisation was carried out using DAVID (version 6.8), Ingenuity Pathway Analysis—IPA (version 1.1, winter 2018 release) and PANTHER (version 13.1) (Kramer et al., 2014; Mi et al., 2017). KEGG pathways were selected by choosing pathways that had the highest number of genes identified in the ChIP-seq data and had an FDR < 0.05.

### Integration of GWAS Data

GWAS data for genetic susceptibility to *M. bovis* infection previously generated by Richardson et al. (2016) were analysed to determine if subsets of SNPs selected according to their distance to H3K4me3 and Pol II active loci were enriched for significant GWAS hits. The nominal *P* values used in this study were generated using single SNP regression analysis in a mixed animal model as described previously (Richardson et al., 2016). In summary, high-density genotypes (*n* = 597,144) of dairy bulls (*n* = 841) used for artificial insemination were associated with deregressed estimated breeding values for bTB susceptibility that had been calculated from epidemiological information on 105,914 daughters and provided by the Irish Cattle Breeding Federation (ICBF). In this study, the significance of the distribution of SNP nominal *P* values (from Richardson et al., 2016) within and up to 100 kb up- and downstream to genes identified as having differential H3K4me3 and Pol II activity on bTB susceptibility was estimated in R using *q* value (FDRTOOL) and permutation analysis (custom scripts). A total of 1,000 samplings (with replacement) from the HD GWAS *P* value data set (*n* = 597,144) representing the size of each of selected SNP subsets were generated. The *q* values for each SNP *P* value subset and all its permuted equivalents were calculated using the FDRTOOL library in R. The subsequent significance level (*P*_perm_) assigned to each of the SNP subsets was equivalent to the proportion of permutations in which at least the same number of *q* values < 0.05 as the SNP subset were obtained, i.e. by chance.

## Results

### *M. bovis* Infection Induces Trimethylation of H3K4 at Key Immune Function Related Loci in Bovine Alveolar Macrophages

Previous studies have shown that bAM undergo extensive gene expression reprogramming following infection of *M. bovis* (Nalpas et al., 2015; Malone et al., 2018), with almost one half of the detectable transcriptome exhibiting significant differential expression within bovine macrophages 24 h after infection (Nalpas et al., 2015). Changes of this magnitude are comparable to those observed in previous experiments that have examined the chromatin remodelling that accompanies mycobacterial infection of macrophages, where trimethylation of lysine 4 of Histone H3 (H3K4me3) was shown to correlate with active transcription (Bouttier et al., 2016; Arts et al., 2018).

**Figure 1:**
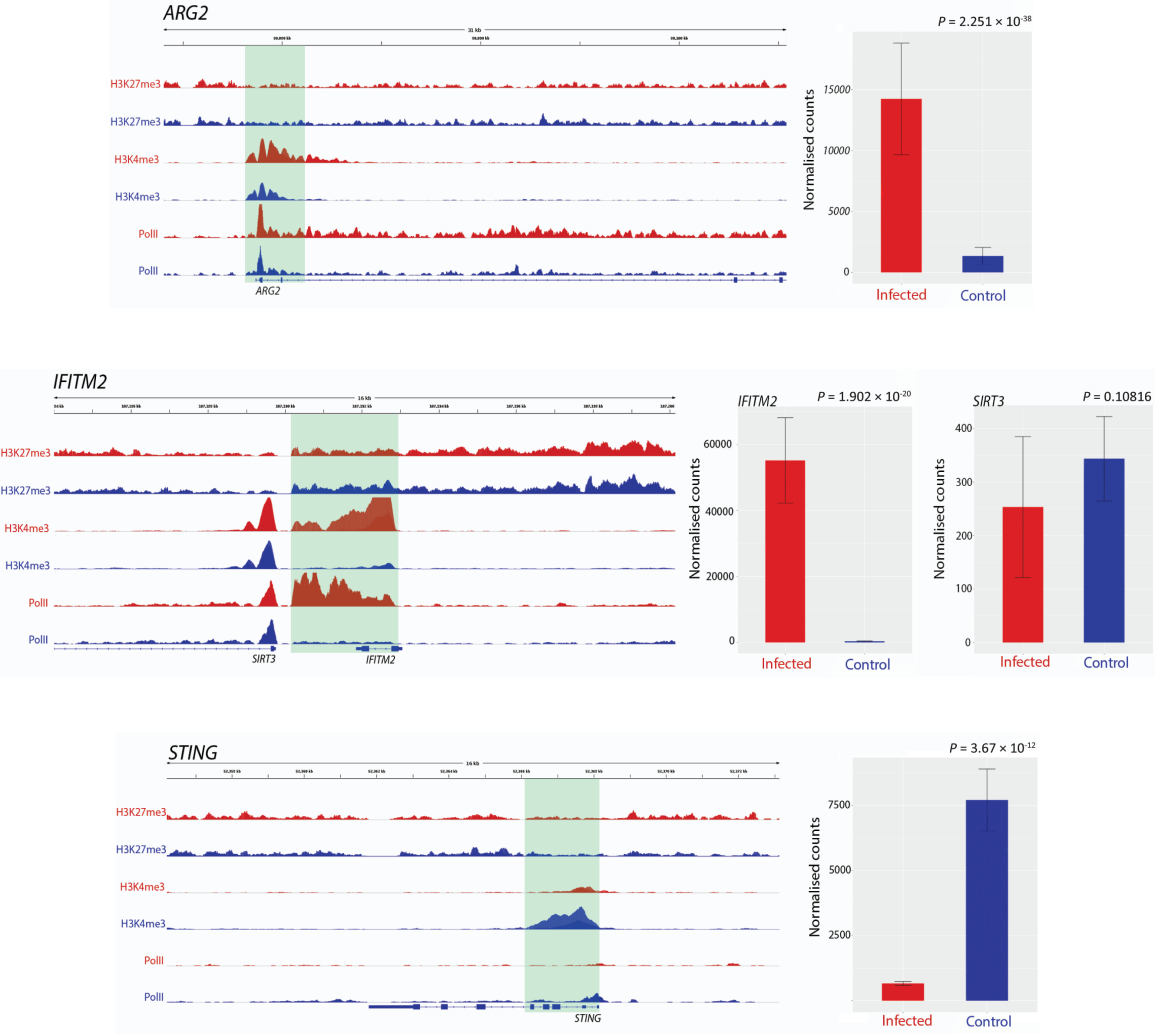
Track visualization of *M. bovis* induced H3K4me3 and Pol II occupancy with relative change in expression at three immune response associated genes. Examples of signal tracks illustrating peaks of H3K27me3 (top two tracks), H3K4me3 (middle two tracks) and Pol II (bottom two tracks) in infected (red) and non-infected (blue) bovine alveolar macrophages, with the bovine reference genome on the bottom of each panel reading left to right. Accompanying each track image is the expression of the corresponding gene, with normalised counts of infected cells in red and control in blue. The *ARG2* gene exhibited an increase in H3K4me3 at 24 hpi as evidenced by the larger red H3K4me3 and red Pol II peaks. The *IFITM2* gene also exhibited larger H3K4me3 and Pol II peaks in infected samples; however, in contrast to this, *SIRT3*, which is located ~20 kb upstream from *IFITM2* gene, had no significant change in either peak. *TMEM173* (aka *STING*) exhibits an opposite pattern to most genes identified as having differential H3K4me3, where a larger peak is observed in control samples rather than infected.

We used ChIP-seq to examine histone modification changes that occur after *M. bovis* infection of bAM from sex- and aged-matched Holstein-Friesian cattle. The aim was to determine genome-wide changes in the distribution of H3K4me3 and H3K27me3, and Pol II occupancy at the response genes (Sims et al., 2003). Differential peaks between conditions were called, compared and visualised with IGV to determine where differences in H3K4me3, H3K27me3 and Pol II occupancy occur between control and infected bAM (**Figure 1**). ChIP-seq peaks are defined as areas of the genome enriched by read counts after alignment to the reference genome.

Peak differences for H3K4me3 occurred at multiple locations across the genome and were estimated by the fold enrichment of a peak normalised against input control DNA that had not undergone antibody enrichment. Differential peaks in each condition were defined by several criteria: 1) the fold enrichment of each peak had to be larger than 10 in at least one condition (Landt et al., 2012); 2) the identified peaks had a *P*-value cut off of 0.05; 3) the peaks being compared in each condition were no more than 500 bp up- and downstream of each other; 4) the peaks were classified as different using log-likelihood ratios and affinity scores with MACS2 and diffBind, respectively; and 5) visual inspection of the tracks of the peaks confirmed the computationally determined differences in each condition.

**Figure 2:**
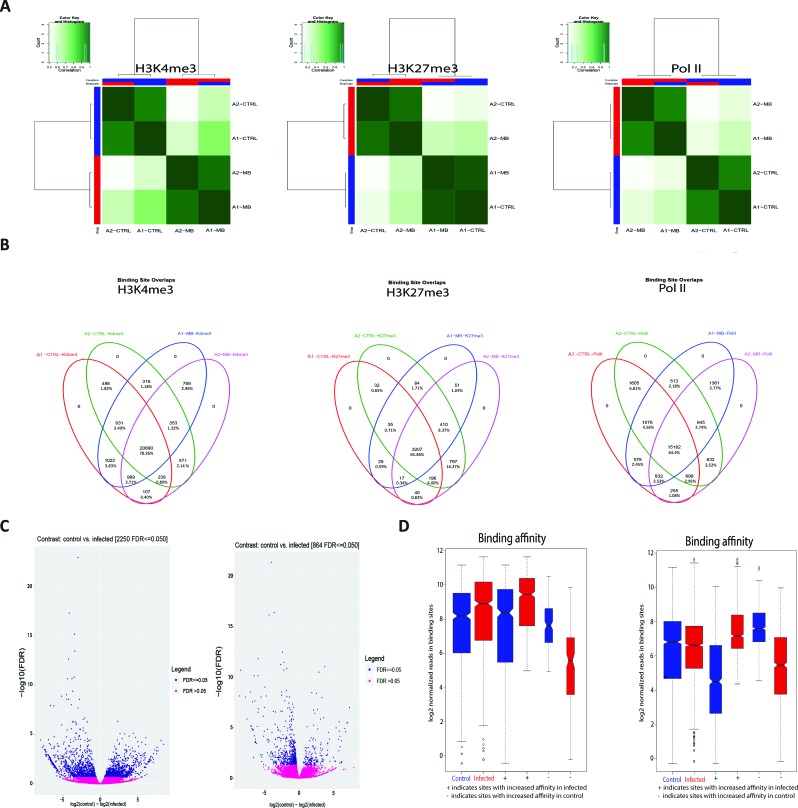
*M. bovis* induced histone modifications occur genome wide at key immune loci. **(A)** Correlation heatmaps of differential peaks for H3K4me3, H3K27me3 and Pol II. Every peak location that is not consistent between each animal in each condition (i.e. a peak only occurs in the control group) is compared to determine if these inconsistent peaks are correlated with the animal or the condition. The differential peaks in H3K4me3 and Pol II correlate highly with condition, whereas there were no significant global differences in the distribution of H3K27me3. **(B)** Venn diagrams of differential peaks for H3K4me3, H3K27me3 and Pol II. Each condition shares most peaks. Where differences occur at TSS of genes, these genes are frequently associated with immune function. **(C)** Volcano plots of differential peaks for H3K4me3 and Pol II. The y-axis shows significance as FDR and the x-axis indicates increase in affinity for control (left) and infected (right). Significant sites are denoted in blue. **(D)** Boxplots of differential peaks for H3K4me3 and Pol II. Infected bAM are shown in red and control bAM are shown in blue. The left two boxes of each plot show distribution of reads over all differentially bound sites in the infected and control groups. The middle two boxes of each plot show the distribution of reads in differentially bound sites that increase in affinity in the control group. The far-right boxes in each plot show the distribution of reads in differentially bound sites that increase in affinity in the infected group.

Peaks that occurred in a sample indicate that H3K4me3 and Pol II are highly correlated with condition (**Figure 2A**); this demonstrates that the differences in H3 modifications are a result of infection rather than genomic differences between animals. **Figure 2B** further illustrates this, with the overlap in enriched peaks for H3K4me3 and Pol II being greater between condition than animal, i.e. the common number of H3K4me3 peaks between animal 1 control and animal 1 infected is 316 and the common number of H3K4me3 peaks between animal 1 infected and animal 2 infected is 798. **Figures 2C, D** illustrate that the distribution of the peaks, or sites with increased binding affinity, is differentially distributed between control and infected for both H3K4me3 and Pol II. Binding site affinity for H3K27me3 showed no significant differences between the control and infected groups for any genes. Analysis of genome-wide H3K4me3 revealed significant peak differences between control and infected samples at multiple sites in the genome under these criteria, with some of these differences occurring at the transcriptional start site of 233 genes. (**Figures 2A–D** and **Supplementary Figure 4**). **Supplementary Figure 1** demonstrates that the differences in H3K4me3 and Pol II peaks are minor, with cells from both conditions sharing most peaks and differing by only 1.8–2.95% in peaks across the genome. Principal component analysis (PCA) of the H3K4me3 mark and Pol II data indicated that these H3K4me3 and Pol II peak differences are strongly associated with *M. bovis* infection of bAM (**Supplementary Figure 3**).

### Changes in H3K4me3 Are Accompanied by Immune Related Transcriptional Reprogramming

**Figure 3:**
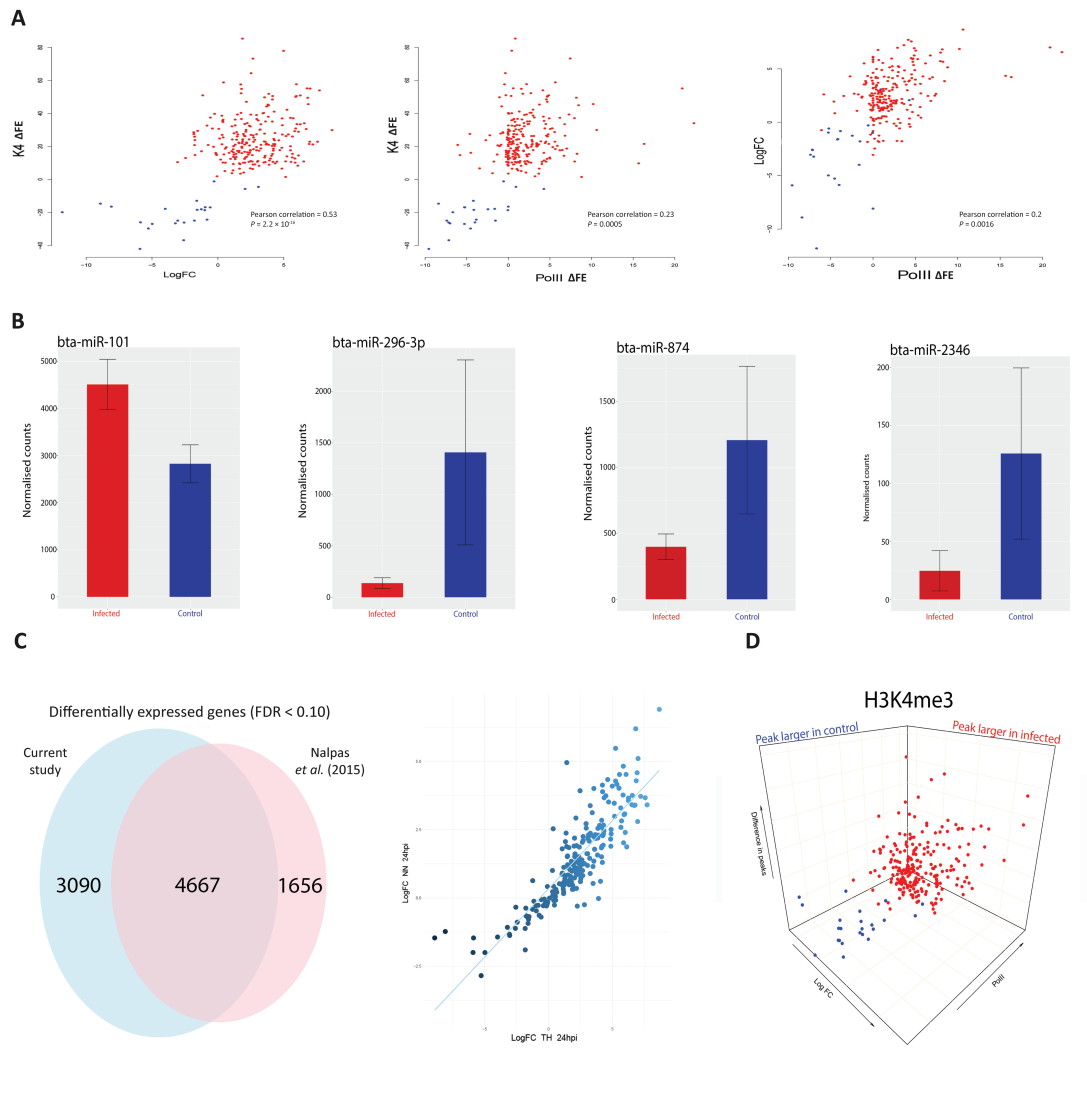
H3K4me3 is accompanied by functional changes in Pol II occupancy, gene expression and gene regulation. **(A)** Scatter plots of H3K4me3 against Pol II occupancy and gene expression. The first plot is the difference of peaks for H3K4me3 between conditions. The values on the y-axis correspond to the difference in fold enrichment (ΔFE) of each peak at each gene between the control and infected groups. A positive value (x) means that the peak was x-fold enriched in the infected cells (red dots) compared to the control cells. Negative values correspond to the peaks being larger in the control (blue dots), indicating a decrease or total depletion of the peak in the infected cells. The x-axis represents the log_2_FC for each of the 232 genes, with each gene as a single data point. The second plot also has H3K4me3 on the y-axis but with peak differences in Pol II on the x-axis, with negative and positive values corresponding to greater occupancy in the control and infected samples, respectively. The final plot shows log_2_FC relative to Pol II occupancy. **(B)** Plots of normalised miRNA-seq counts. Each plot represents the normalised counts of a miRNA that was detected as exhibiting differential expression. Bta-miR-101 interacts with *ARG2*, bta-miR-296-3p with *TMEM173* (aka *STING*), bta-miR-874 with *BCL2A1* and bta-miR-2346 with *STAT1*. Red bars indicate infected and blue represent control samples. **(C)** Correlation and Venn diagram for both RNA-seq studies. The x-axis of the scatter plot represents the log_2_FC for each of the 232 genes from this study and the y-axis represents the log_2_FC for each of the 232 genes from the previous study (Nalpas et al., 2015). The Venn diagram shows the global overlap of differentially expressed genes from both studies with an FDR cut-off < 0.1. **(D)** 3-D plots for all three data sets. A combination of all three scatter plots from **Figure 3A**. Data points are genes. Blue genes are those that exhibited greater H3K4me3 in control bAM; red exhibited greater H3K4me3 in infected bAM.

Previous studies have shown that increased H3K4me3 is frequently accompanied by an increase in Pol II occupancy and elevated expression of proximal genes (Clouaire et al., 2012; Barski et al., 2017). In the present study, we observed that H3K4me3 is accompanied by an increase in Pol II occupancy (**Figure 1** and **Supplementary Figure 4**). For a small number of genes (24 out of 233) where the H3K4me3 peak was larger in the control than the infected samples, Pol II occupancy was greater in control bAM for 20 genes (83.3%) and greater in infected bAM for 3 genes (12.5%). Conversely, where the H3K4me3 peak was larger in the infected bAM, Pol II occupancy was greater in the infected samples for 127 genes (60.4%) and greater in the control bAM for 14 genes (6.6%). The remaining 60 genes (25%) did not exhibit H3K4me-associated Pol II occupancy in either control or infected samples. **Figure 3A** illustrates this trend, showing that Pol II occupancy normally accompanies H3K4me3.

To establish if H3K4me3 mark patterns were correlated with changes in gene expression, control non-infected bAM and bAM infected *M. bovis* AF2122/97 from four animals 24 hpi (including the two animals used for ChIP-seq) were used to generate eight RNA-seq libraries. RNA-seq analysis revealed 7,757 differentially expressed genes (log_2_FC > 0: 3,723 genes; log_2_FC < 0: 4,034 genes; FDR < 0.1). Of the 233 genes identified in the ChIP-seq analysis, 232 (99.6%) were differentially expressed under these criteria (see **Supplementary Information File 2**). Of the genes that exhibited H3K4me3 peaks that were larger in the infected bAM, 21 (10%) were downregulated and 189 (90%) were upregulated. Of the genes that exhibited larger H3K4me3 peaks in the control group, 22 (91.6%) were downregulated and 2 (8.4%) were upregulated (**Figure 3A**). This pattern of directional gene expression correlating with H3K4me3 for the control and infected samples is consistent with the literature (Clouaire et al., 2012; Barski et al., 2017).

Existing published RNA-seq data generated by our group using *M. bovis*-infected (*n* = 10) and control non-infected bAM (*n* = 10) at 24 hpi (Nalpas et al., 2015) were also examined in light of the results from the present study. For the 232 genes identified here, a Pearson correlation coefficient of 0.85 was observed for two data sets (**Figure 3C**), thus demonstrating that gene expression differences between *M. bovis*-infected and control non-infected bAM are consistent across experiments, even where samples sizes are markedly different.

### Transcriptional Reprogramming Is Coupled With Differential microRNA Expression

We have previously demonstrated that differential expression of immunoregulatory microRNAs (miRNAs) is evident in bAM infected with *M. bovis* compared to non-infected control bAM (Vegh et al., 2013; Vegh et al., 2015). To investigate the expression of miRNA in bAM used for the ChIP-seq analyses, miRNA was extracted and sequenced from the samples used for the RNA-seq analysis. Twenty-three differentially expressed miRNAs were detected at 24 hpi (log_2_FC > 0: 13; log_2_FC < 0: 10; FDR < 0.10). Of the 232 genes identified in the ChIP-seq/RNA-seq analysis, 93 are potential targets for the 23 differentially expressed miRNAs (**Supplementary Information File 3**). Further examination revealed that multiple immune genes, such as *BCL2A1* (bta-mir-874), *ARG2* (bta-mir-101)*, TMEM173* (aka *STING*) (bta-mir-296-3p) and *STAT1* (bta-mir-2346), are potential regulatory targets for these miRNAs (**Figure 3B**). This observation therefore supports the hypothesis that miRNAs function in parallel with chromatin modifications to modulate gene expression in response to infection by *M. bovis*.

### Integration of ChIP-Seq and RNA-Seq Data

The H3K4me3, Pol II, H3K27me3 ChIP-seq data and the RNA-seq data were subsequently integrated to evaluate the relationship between histone modifications and gene expression changes. Three-dimensional plots were generated to visualise the global differences between H3K4me3, Pol II and gene expression in infected and non-infected bAM (**Figure 3D**). These plots show that reduction of H3K4me3 in infected cells is associated with a decrease in gene expression and an absence of Pol II occupancy. Genome-wide H3K27me3 was also investigated to determine whether methylation of this residue was altered in response to *M. bovis* infection and if it was related to gene expression. No significant differences for H3K27me3 between control and infected bAM were detected, indicating that repression of gene expression through H3K27me3 does not play a role in the bAM response to *M. bovis* at 24 hpi. However, **Supplementary Figure 2** indicates that the presence of a H3K27me3 peak in both control and infected cells at the TSS of a H3K4me3 enriched gene correlated well with a lower or complete lack of Pol II occupancy.

### Pathway Analysis Reveals H3K4me3 Marks Are Enriched for Key Immunological Genes

**Figure 4:**
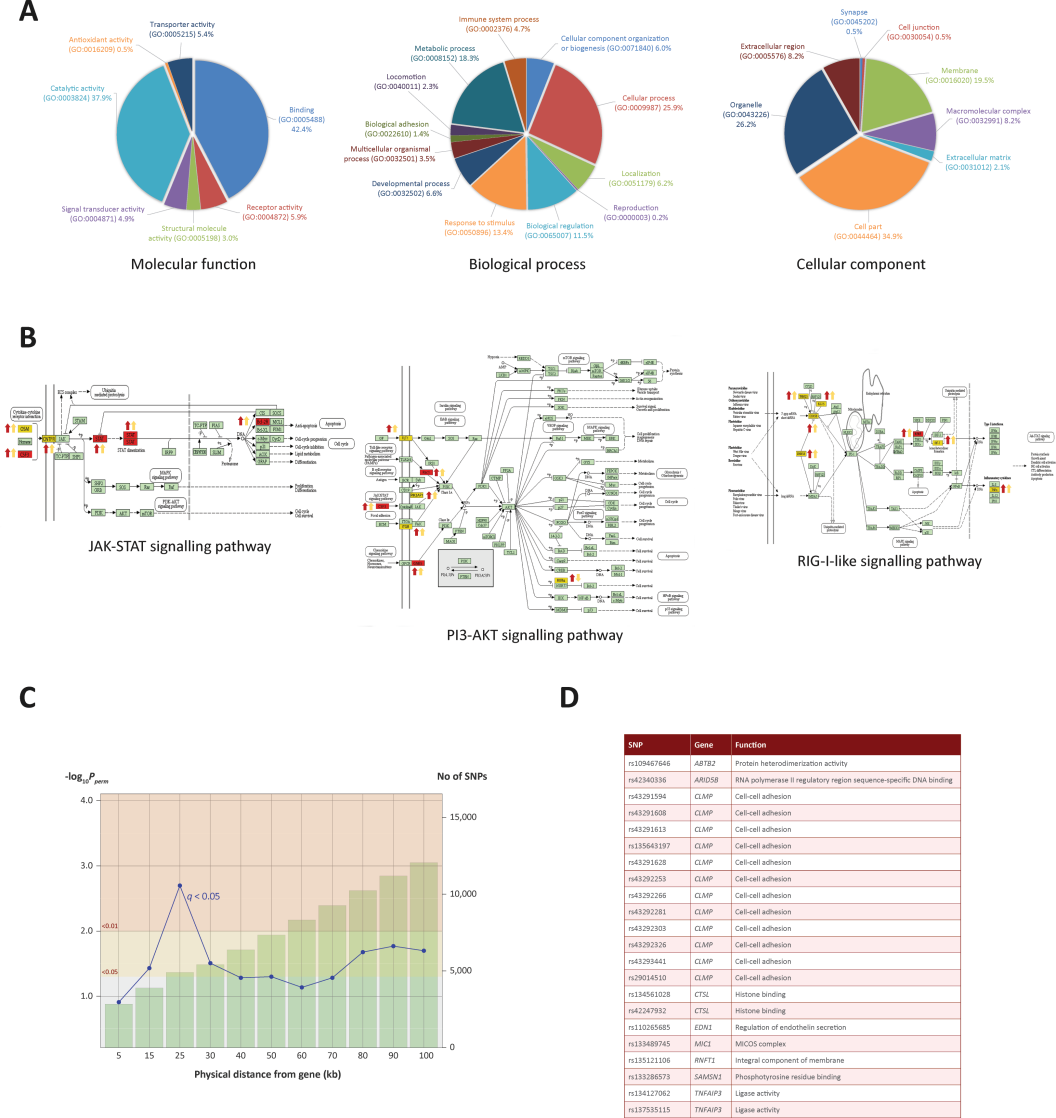
Gene ontology enrichment and pathway analysis. **(A)** Gene ontology pie charts generated through PANTHER pathway analysis; 232 genes cluster by gene ontology under three main categories: *Biological process*, *Cellular component* and *Molecular function*. **(B)** KEGG pathway images containing genes identified from the ChIP-seq and RNA-seq analysis. Gene symbols coloured in yellow were identified in the ChIP-seq and RNA-seq analysis. Gene symbols coloured in red were also targeted by one or more differentially expressed miRNAs. Up or down red arrows indicate greater H3K4me3 in infected or control, respectively. Up or down yellow arrows indicate log2FC increase or decrease of the associated gene, respectively. **(C)** Line graph showing different genomic ranges from genes that are enriched for significant SNPs from GWAS data for bTB resilience. The bars represent the number of SNPs that occupy each range from each ChIP-seq enriched gene, with more SNPs correlating with a greater distance. The blue plotted line represents the negative log_10_ probability that the significant SNPs found at each distance at 0.05 FDR *q* value are significant by chance, with SNPs at 25 kb exhibiting the lowest probability. The null SNP *P* value distribution for each data point was generated from 1,000 permutations of random SNPs corresponding to the number of SNPs observed in a particular genomic range. **(D)** Genes enriched for SNPs significantly associated with resilience to *M. bovis* infection. SNP IDs and functional information obtained from the GeneCards^®^ database (Stelzer et al., 2016) are also shown.

To identify biological pathways associated with genes identified through the ChIP-seq analyses, we integrated the ChIP-seq, RNA-seq and miRNA-seq data, which generated a panel of 93 genes that overlapped across each of the three data sets. Pathway analyses were carried out using three software tools: Ingenuity Pathway Analysis (IPA), Panther and DAVID (Thomas et al., 2003; Huang da et al., 2009; Kramer et al., 2014). IPA revealed an association with *respiratory illness* and the *innate immune response* (**Supplementary File 2**). Panther was used to examine the GO categories of the 232 genes (**Figure 4A**); this revealed enrichment for metabolic processes, response to stimuli and cellular processes, indicating that increased H3K4me3 in response to *M. bovis* infection occurs at TSS of genes associated with the immune response and at genes encoding key components of internal macrophage cellular regulation. GO unifies genes based on their gene and gene product attributes, which represents a useful method of identifying the families of gene functions for a given enriched gene set such as the one summarised in **Figure 4A** (The Gene Ontology Consortium, 2019).

The final part of the pathway analysis was performed using DAVID (Huang da et al., 2009). DAVID uses a list of background genes and query genes (in this case the 232 common genes across data sets) and identifies enriched groups of genes with shared biological functions. The DAVID analysis demonstrated that the 232 genes are involved in several signalling pathways, including the PI3K/AKT/mTOR, JAK-STAT and RIG-I-like signalling pathways (**Figure 4B** and the top 10 pathways are detailed in **Supplementary Information File 3**).

### GWAS Integration Prioritises Bovine SNPs Associated With Resilience to *M. bovis* Infection

Previous work used high-density SNP (597,144 SNPs) data from 841 Holstein-Friesian bulls for a GWAS to detect SNPs associated with susceptibility/resistance to *M. bovis* infection (Richardson et al., 2016). Using a permutation-based approach to generate null SNP distributions, we leveraged these data to show that genomic regions within 100 kb up- and downstream of each of the 232 genes exhibiting differential H3K4me3 ChIP-seq peaks are significantly enriched for additional SNPs associated with resilience to *M. bovis* infection.

In total, 12,056 SNPs within the GWAS data set were located within 100 kb of the 232 H3K4me3 genes. Of these SNPs, up to 26 were found to be significantly associated with bTB susceptibility, depending on the distance interval of each gene. Interestingly, 22 SNPs found within 25 kb of 11 genes were found to be most significant at *P* and *q* values < 0.05, with declining significance of association as the region extended beyond 25 kb (**Figures 4C**, **4D** and **Supplementary File 3**). Significant SNPs were detected in proximity to the following genes: *SAMSN1, CTSL, TNFAIP3, CLMP, ABTB2, RNFT1, MIC1, MIC2, EDN1* and *ARID5B*, all of which had significant differential enrichment of H3K4me3.

## Discussion

### H3K4me3 Mark Occurs at Key Immune Genes

Our study has generated new information regarding host–pathogen interaction during the initial stages of *M. bovis* infection. We demonstrate that chromatin is remodelled through differential H3K4me3 and that Pol II occupancy is altered at key immune genes in *M. bovis*-infected bAM. This chromatin remodelling correlates with changes in the expression of genes that are pivotal for the innate immune response to mycobacteria (Nalpas et al., 2015; Alcaraz-Lopez et al., 2017; Malone et al., 2018). Our work supports the hypothesis that chromatin modifications of the host macrophage genome play an essential role during intracellular infections by mycobacterial pathogens (Cheng et al., 2014; LaMere et al., 2016).

The top pathways identified were the JAK-STAT signalling pathway, the PI3K/AKT/mTOR signalling pathway and the RIG-I-like receptor signalling pathway. In mammals, the JAK-STAT pathway is the principal signalling pathway that modulates expression of a wide array of cytokines and growth factors, involved in cell proliferation and apoptosis (Rawlings et al., 2004). The JAK-STAT signalling pathway and its regulators are also associated with coordinating an effective host response to mycobacterial infection (Manca et al., 2005; Cliff et al., 2015). Two JAK-STAT associated stimulating factors and a ligand receptor that exhibited increased H3K4me3 marks in infected samples were encoded by the *OSM*, *CSF3* and *CNTFR* genes, respectively (Marino and Roguin, 2008; Pastuschek et al., 2015)*. OSM* has previously been shown to be upregulated in cells infected with either *M. bovis* or *M. tuberculosis* (O'Kane et al., 2008; Nalpas et al., 2015; Polena et al., 2016). Our work shows that this increased expression in response to mycobacteria is facilitated by H3K4me3-mediated chromatin accessibility. The protein encoded by *CSF3* has also been implicated as an immunostimulator in the response to mycobacterial infection due to its role in granulocyte and myeloid haematopoiesis (Martins et al., 2010). *CNTFR* encodes a ligand receptor that stimulates the JAK-STAT pathway and shows increased expression in other studies of mycobacterial infection (Nalpas et al., 2015; Malone et al., 2018). Following stimulation of JAK through ligand receptor binding, *STAT1* expression is increased. STAT1, a signal transducer and transcription activator that mediates cellular responses to interferons, cytokines and growth factors, is a pivotal JAK-STAT component and a core component in the response to mycobacterial infection (Tsumura et al., 2012). Here, the TSS of *STAT1* was associated with an increased deposition of H3K4me3. Interestingly, upregulation of *STAT1* was associated with a downregulation of bta-miR-2346, predicted to be a negative regulator of STAT1 (see **Supplementary Information File 3**). Overall, these results show that major components of the JAK-STAT pathway undergo chromatin remodelling mediated *via* H3K4me3, thereby facilitating activation and propagation of the JAK-STAT pathway through chromatin accessibility.

Key genes encoding components of the PI3K/AKT/mTOR pathway, such as *IRF7*, *RAC1* and *PIK3AP1*, were also identified as having increased H3K4me3 in *M. bovis* infected macrophages. PI3K/AKT/mTOR signalling contributes to a variety of processes that are critical in mediating aspects of cell growth and survival (Yu and Cui, 2016). Phosphatidylinositol-3 kinases (PI3Ks) and the mammalian target of rapamycin (mTOR) are integral to coordinating innate immune defences (Weichhart and Saemann, 2008). The PI3K/AKT/mTOR pathway is an important regulator of type I interferon production *via* activation of the interferon-regulatory factor 7, IRF7. RAC1 is a key activator of the PI3K/AKT/mTOR pathway and, in its active state, binds to a range of effector proteins to regulate cellular responses such as secretory processes, phagocytosis of apoptotic cells and epithelial cell polarisation (Yip et al., 2007). In addition, *in silico* analysis of our differentially expressed miRNAs predicted that several miRNAs, such as bta-miR-1343-3p, bta-miR-2411-3p and bta-miR-1296, regulate *RAC1*. *PIK3AP1* expression was also increased, in line with previous mycobacterial infection studies (Nalpas et al., 2015; Malone et al., 2018). Hence, as observed with the JAK-STAT pathway, H3K4me3 at these key PI3K/AKT/mTOR pathway genes acts to regulate the innate response to mycobacterial infection. In addition, the RIG-I-like receptor signalling pathway was also highlighted by the ChIP-seq, RNA-seq and miRNA-seq integrative analyses. Genes encoding multiple components of this pathway, such as *TRIM25*, *ISG15*, *IRF7* and *IKBKE*, were enriched for H3K4me3 and Pol II occupancy in *M. bovis*-infected bAM. The RIG-I-like receptor signalling pathway activates transcription factors that regulate production of type I interferons (Loo and Gale, 2011) and our results demonstrate that activation of this pathway in *M. bovis*-infected bAM is driven, to a large extent, by reconfiguration of the host chromatin.

H3K4me3 enriched loci are also flanked by genomic polymorphisms associated with resilience to *M. bovis* infection. Integration of our data with GWAS data from 841 bulls that have robust phenotypes for bTB susceptibility/resistance revealed 22 statistically significant SNPs within 25 kb of 11 H3K4me3 enriched genes. Statistical significance was determined if the newly permuted *q* values of every SNP found in proximity to each of the H3K4me3 enriched genes is unique to the observed set, when compared to 1,000 random sets of SNPs from the same GWAS (i.e. if significant *q* values of the same value or less occur with the same or greater frequency in randomised SNP sets, the observed SNPs are not deemed to be statistically significant). Most of these genes are involved in host immunity, with *CTSL, TNFAIP3* and *RNFT1* directly implicated in the human response to *M. tuberculosis* infection (Nepal et al., 2006; Silver et al., 2009; Meenu et al., 2016). The reprioritisation of genomic regions and array-based SNPs using integrative genomics approaches will be relevant for genomic prediction and genome-enabled breeding and may facilitate fine-mapping efforts and the identification of targets for genome editing of cattle resilient to bTB.

### H3K4me3 Deposition at Host Macrophage Genes and Immunological Evasion by *M. bovis*

The present study has revealed elevated H3K4me3 deposition and Pol II occupancy at key immune genes that are involved in the innate response to mycobacterial infection. In addition, we also identified several immune genes that had differential H3K4me3 and expression, where the expression change may be detrimental to the ability the host macrophage to clear infection. An example of this is *ARG2*, which exhibited increased H3K4me3 deposition, Pol II occupancy and expression (Log_2_FC = 3.415, *P*_adj_ = 7.52 ×10^-16^) in infected cells. However, it is also interesting to note that the integrated expression output of *ARG2* may also be determined by the bta-miR-101 miRNA, a potential silencer of *ARG2* expression, which was observed to be upregulated in infected cells. Elevated levels of arginase 2, the protein product of the *ARG2* gene, have previously been shown to shift macrophages to an M2 phenotype (Lewis et al., 2011; Hardbower et al., 2016), which is anti-inflammatory and exhibits decreased responsiveness to IFN-γ and decreased bactericidal activity (Huang et al., 2015). Hence, it may be hypothesised that *M. bovis* infection triggers H3K4me3 deposition at the TSS of *ARG2* to drive an M2 phenotype and generate a more favourable niche for the establishment of infection. Like *ARG2*, increased expression of *BCL2A1* in *M. bovis*-infected bAM may also facilitate development of a replicative milieu for intracellular mycobacteria. Increased expression of *BCL2A1* is associated with decreased macrophage apoptosis (Vogler, 2012), which would otherwise restrict replication of intracellular pathogens.

In comparison to control non-infected bAM, the *TMEM173* (aka *STING*) gene exhibited substantially decreased expression in *M. bovis*-infected bAM (Log_2_FC = −3.225, *P*_adj_ = 8.64 ×10^-11^). *TMEM173* encodes transmembrane protein 173, which drives interferon production and as such is a major regulator of the innate immune response to viral and bacterial infections, including *M. bovis* and *M. tuberculosis* (Manzanillo et al., 2012; McNab et al., 2015; Malone et al., 2018). Downregulation of *TMEM173* indicates that *M. bovis* can actively reduce or block methylation of H3K4 at this gene in infected macrophages, thereby enhancing intracellular survival of the pathogen. In this regard, we have recently shown that infection of bAM with *M. tuberculosis*, which is attenuated in cattle, causes increased *TMEM173* expression compared to infection with *M. bovis* (Malone et al., 2018).

The molecular mechanisms that pathogens employ to manipulate the host genome to subvert or evade the immune response are yet to be fully elucidated. Hijacking the host’s own mechanisms for chromatin modulation is one potential explanation that has garnered attention in recent years (Hamon and Cossart, 2008; Rolando et al., 2015). These modulations of the host chromatin in bAM may be mediated through *M. bovis*-derived signals transmitted through bacterial metabolites, RNA-signalling or secreted peptides (Silmon de Monerri and Kim, 2014; Sharma et al., 2015; Yaseen et al., 2015; Woo and Alenghat, 2017).

## Conclusions

Elucidation of the mechanisms used by pathogens to establish infection, and ultimately cause disease, requires an intimate knowledge of host–pathogen interactions. Using transcriptomics and epigenomics, we have identified altered expression of major host immune genes following infection of primary bovine macrophages with *M. bovis*. We have shown that reprogramming of the alveolar macrophage transcriptome occurs mainly through increased deposition of H3K4me3 at key immune function genes, with additional gene expression modulation *via* miRNA differential expression. This modulation of gene expression drives a shift of the macrophage phenotype towards the more replication-permissive M2 macrophage phenotype. We have also identified that alveolar macrophages infected with *M. bovis* exhibit differentially expressed genes (in regions with modified chromatin) that are enriched for significant SNPs from GWAS data for bTB resilience. Finally, our results support the emerging concept that pathogens can hijack host chromatin, through manipulation of H3K4me3, to subvert host immunity and to establish infection.

## Data Availability Statement

The ChIP-seq, RNA-seq and microRNA-seq data sets have been submitted to the NCBI Gene Expression Omnibus (GEO) with accession number GSE116734.

## Ethics Statement

All animal procedures were performed according to the provisions of Statutory Instrument No. 543/2012 (under Directive 2010/63/EU on the Protection of Animals used for Scientific Purposes). Ethical approval was obtained from the University College Dublin Animal Ethics Committee (protocol number AREC-13-14-Gordon).

## Author Contributions

Project conceptualisation: AO’D, DM and TH. Software and formal analysis: TH and MM. Investigation: AO’D, DV and JB. Resources: DM, SG and DV. Data curation: TH. Writing—original draft: TH, AO’D and DM. Writing—review and editing: D.V., SG and MM. Supervision and project administration: DM and AO’D. Funding acquisition: DM, SG, DV and AO’D.

## Funding

This study was supported by Science Foundation Ireland (SFI) Investigator Programme Awards to DM and SG (grant nos. SFI/08/IN.1/B2038 and SFI/15/IA/3154); a European Union Framework 7 Project Grant to DM (no: KBBE-211602-MACROSYS); an EU H2020 COST Action short-term scientific mission (STSM) grant to AO’D (reference code: COST-STSM-ECOST-STSM-CA15112-050317-081648); a University of Edinburgh Chancellor’s Fellowship to DV; and BBSRC Institute Strategic Grant funding to the Roslin Institute (grant nos. BBS/E/D/10002070 and BBS/E/D/20002172). The funding agencies had no role in the study design, collection, analysis and interpretation of data, and no role in writing the manuscript.

## Conflict of Interest

The authors declare that the research was conducted in the absence of any commercial or financial relationships that could be construed as a potential conflict of interest.

## Abbreviations

bAM: bovine alveolar macrophage/s; bTB: bovine tuberculosis; ChIP: chromatin immunoprecipitation; FDR: false discovery rate; GWAS: genome-wide association study; H3K27me3: histone H3 lysine 27 tri-methylation; H3K4me3: histone H3 lysine 4 tri-methylation; hpi: hours post infection; log_2_FC: log_2_ fold change; Pol II: RNA Polymerase II; SNP: single-nucleotide polymorphism; TB: tuberculosis.
